# Publication Trends and Hot Spots of ChatGPT’s Application in the Medicine

**DOI:** 10.1007/s10916-024-02074-y

**Published:** 2024-05-18

**Authors:** Zhi-qiang Li, Xue-feng Wang, Jian-ping Liu

**Affiliations:** 1 Centre for Evidence-based Chinese Medicine, Beijing University of Chinese Medicine, Beijing, 100029 China; 2 The National Research Center in Complementary and Alternative Medicine (NAFKAM), Department of Community Medicine, Faculty of Health Science, UiT the Arctic University of Norway, Tromsø, Norway

**Keywords:** ChatGPT, Bibliometric analysis, Clustering analysis, Citespace

## Abstract

This study aimed to analyze the current landscape of ChatGPT application in the medical field, assessing the current collaboration patterns and research topic hotspots to understand the impact and trends. By conducting a search in the Web of Science, we collected literature related to the applications of ChatGPT in medicine, covering the period from January 1, 2000 up to January 16, 2024. Bibliometric analyses were performed using CiteSpace (V6.2., Drexel University, PA, USA) and Microsoft Excel (Microsoft Corp.,WA, USA) to map the collaboration among countries/regions, the distribution of institutions and authors, and clustering of keywords. A total of 574 eligible articles were included, with 97.74% published in 2023. These articles span various disciplines, particularly in Health Care Sciences Services, with extensive international collaboration involving 73 countries. In terms of countries/regions studied, USA, India, and China led in the number of publications. USA ot only published nearly half of the total number of papers but also exhibits a highest collaborative capability. Regarding the co-occurrence of institutions and scholars, the National University of Singapore and Harvard University held significant influence in the cooperation network, with the top three authors in terms of publications being Wiwanitkit V (10 articles), Seth I (9 articles), Klang E (7 articles), and Kleebayoon A (7 articles). Through keyword clustering, the study identified 9 research theme clusters, among which “digital health”was not only the largest in scale but also had the most citations. The study highlights ChatGPT’s cross-disciplinary nature and collaborative research in medicine, showcasing its growth potential, particularly in digital health and clinical decision support. Future exploration should examine the socio-economic and cultural impacts of this trend, along with ChatGPT’s specific technical uses in medical practice.

**Supplementary Information:**

The online version contains supplementary material available at 10.1007/s10916-024-02074-y.

ChatGPT, an advanced large language model built on the Transformer architecture, signifies a remarkable achievement in natural language processing. It can generate responses of nearly human quality for various tasks. Currently, there is several promising avenues for artificial intelligence(AI) in the healthcare domain [1, 2]. Despite the open and inclusive attitude towards AI in the field of medicine, the utilization status of ChatGPT as a medical aid is still unclear, especially considering that over 1000 related articles have been published. Although there have been bibliometric analyses in the early phase of ChatGPT [3, 4], the included data contains a large number of *Letter, Commentary, Editorial, and Correspondence* type papers. These types of documents typically did not investigate the main trends and topic hotspots in medical research related to ChatGPT, potentially limiting the depth and accuracy of these analyses.Therefore, our goal of this study is thoroughly analyze the current application of ChatGPT in the medical field using bibliometric methods, revealing the development trends, collaboration patterns, and topic hotsport, to provide directions for future development and application.

This bibliometric study used Citespace(V6.2., Drexel University, PA, USA) and Microsoft Excel (Microsoft Corp.,WA, USA) software to quantitatively measure the contributions of ChatGPT in the medical field [5]. Utilizing the Web of Science Core Collection, we executed a targeted search strategy: ((TS=(ChatGPT)) OR TS=(Generative Artificial Intelligence)) OR TS=(GenAI), spanning from January 1, 2000 until Jan 16th, 2024. Criteria for inclusion were: (1) Primary publications on ChatGPT in the medical field, (2) English Language, (3) Document Types is only include original articles and reviews. Exclusion criteria were : (1) Primary papers not relevant to the topics, (2) Publications that were published repeatedly. Two researchers independently conducted data searches, resolving any discrepancies through discussion. Advanced bibliometric tools, including co-cited and cluster analysis, alongside keyword co-occurrence, were applieds (eMethods 1 in Supplement), and we also using the collaboration Index(C-index) to evaluate the collaborative capability of researchers, institutions, or countries/regions with peer-level researchers across continents [6], and the N-index was used to provide a more equitable comparison of researchers working in different disciplines [7] (eMethods 2 in Supplementary).

In the realm of medical applications of ChatGPT, an analysis of 574 publications reveals that 561 (97.74%) were released in the year 2023, significantly indicating the rapid development within this field. Of these, 412 were original articles and 162 were reviews. The top 10 research directions are illustrated in Fig. 1A. The area of Health Care Science Services encompasses 72 publications, which indicates that this field is a significant area of research interest. It is worth noting that each publication could be categorized under one or multiple fields, thereby the total percentage across all research directions could exceed 100%. This is because research is influenced by the extensive data available, which facilitates advanced AI learning and application. This focus aligns with public and professional interest due to these fields**’** close connection with general health concerns. Moreover, the effective application of AI in improving diagnostics, treatment planning, and patient care highlights the potential of these domains for AI integration, making them central to advancements and innovations in medical AI research. A total of 313 distinct journals have accepted and published research papers related to the medical themes of ChatGPT, but each has not yet published more than 40 articles.

**Fig. 1:**
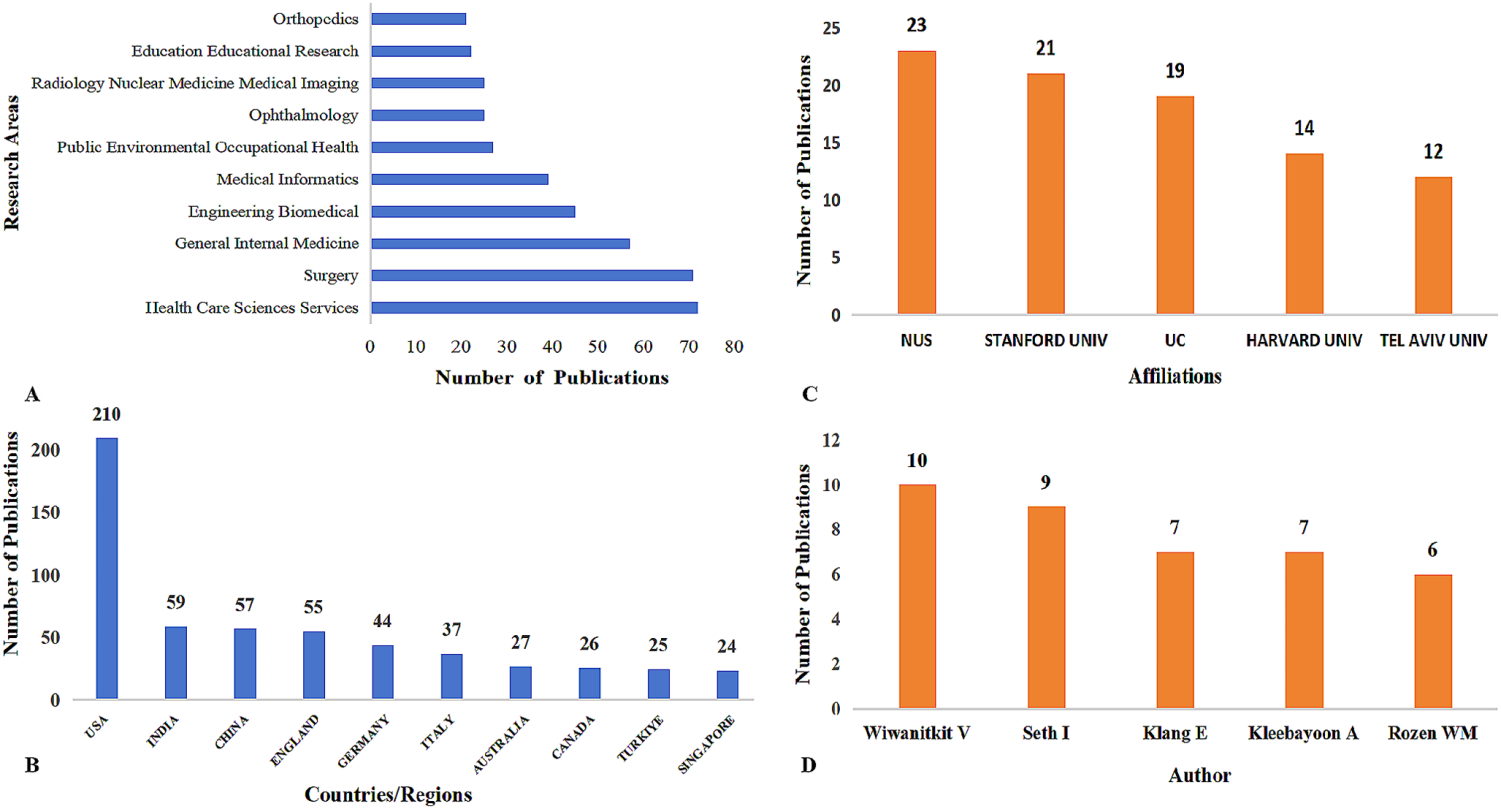
ChatGPT scientific production on medical. (**A**) Number of Research Areas; (**B**) Number of studies per country/region; (**C**) Number of publications of affiliations; (**D**) Number of publications of Authors. *Note* Univ, University. NUS, National Univ of F Singapore; UC, Univ of California System

Figure 2A displays a network map illustrating the international collaboration. A total of 73 countries have contributed to research in this domain, with the top 10 countries in terms of publications shown in Fig. 1B. The USA, India, and China play a leading role with 210 (36.58%), 59 (10.28%), and 57 (9.93%) publications, respectively. Additionally, centrality, an indicator measuring the importance of a node within the network, reflects the frequency at which a node appears on the shortest path between any two points in the network. Nodes with higher centrality indicate the higher research’s influence. Considering both publication volume and top centrality (0.16), USA emerges as the leader in this research domain. Even when considering the C-index of countries/regions, USA significantly surpasses other nations in its collaborative capacity (eTable 1 in Supplementary).

**Fig. 2:**
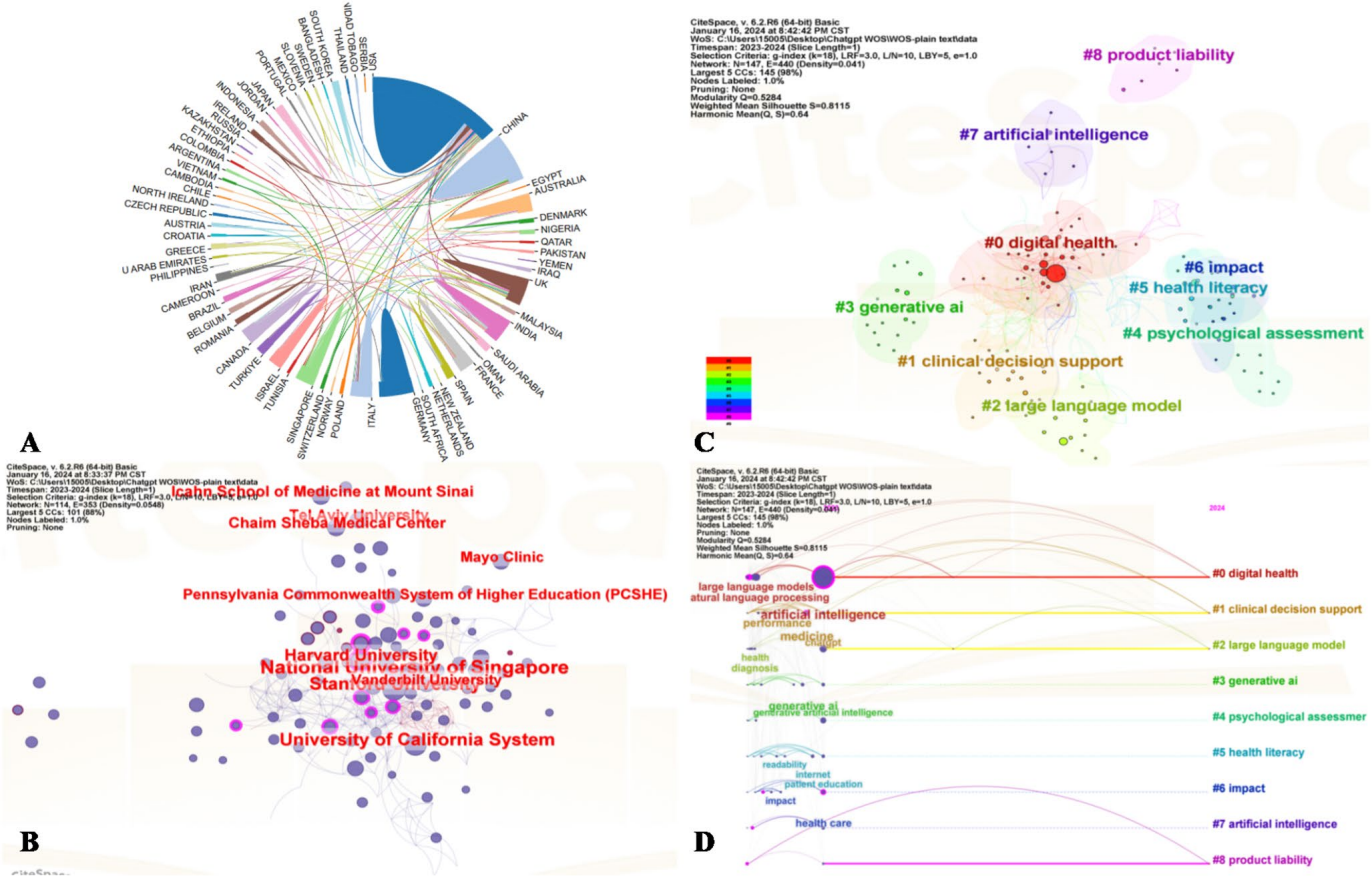
(**A**) Countries/regions co-occurrence network map; (**B**) Institution co-occurrence network map; (**C**) Clustering map of keywords; (**D**) A timeline visualization of the clusters

The institutional co-occurrence network, shows that research teams are relatively dispersed across institutions, highlighting the need for enhanced academic collaboration (Fig. 2B). Notably, the National University of Singapore(NUS) and Harvard University occupy significant positions in the collaboration network in terms of publication volume and centrality, with NUS having the highest publication count and Harvard holding the highest centrality(0.12) (Fig. 1C/Fig. 2B). Similar results can be observed in the C-index for institutions (eTable 2 in Supplementary). Nevertheless, cooperation among research teams is relatively decentralized, and there is still a need to further strengthen international and cross-institutional cooperation. It is heartening to see the presence of Chinese scholars, who are diligently striving to keep pace with scientific frontiers and align with international standards. Co-author network is an important metric for measuring academic collaboration. Since research on the application of ChatGPT in the medical field is still in its early stages, a core network of authors with high influence has not yet formed (Fig. 1D). However, a significant influx of emerging scholars could be observed, reflecting the strong interest of scholars from various countries in applying artificial intelligence to the field of medicine. The top 5 authors by publication volume are Wiwanitkit V (10 papers), Seth I (9 papers), Klang E (7 papers), Kleebayoon A (7 papers), and Rozen WM (6 papers). However, the active author in the field, Wiwanitkit V, shows the highest centrality (0.22). Based on the N-index, it is evident that Wiwanitkit V also demonstrates outstanding performance (0.29). However, in terms of researchers’ C-index, Seth Ishith exhibits the highest collaborative capability (eTable 3 in Supplementary).

Keyword clusters organizes similar keywords together, which represent different research domains, thereby uncovering the core themes and evolution trends of these domains [8]. The size of each cluster indicates the number of papers it contains. In this study, publications related to the application of ChatGPT in the medical field have been divided into 9 main clusters (Fig. 2C), with smaller cluster numbers denoting larger cluster sizes. The largest cluster is #0 digital health. The remaining clusters are #1 clinical decision support, #2 large language model, #3 generative AI, #4 psychological assessment, #5 heath literacy, #6 impact, #7 artificial intelligence, #8 product liability. All cluster maps have a modularity Q value exceeding 0.3 and silhouette scores above 0.8, demonstrating the high reliability of the clustering results. This signals that future research may focus on how to effectively utilize GenAI such as ChatGPT to improve the management and dissemination of health information. Keyword clustering for thematic analysis in bibliometrics, while beneficial, encounters limitations including sensitivity to keywords, overlooking contextual nuances, and the challenge of interpreting results accurately due to the dynamic nature of research trends and the inherent subjectivity in keyword selection. These constraints highlight the need for robust and comprehensive thematic interpretation of analyses of quantitative results. However, Fig. 2D displays a timeline visualization that displays clusters from left to right along horizontal timelines. The legend at the top denotes the publication time, while the clusters are vertically arranged in descending order of size. Notably, the most substantial cluster, digital health, occupies the top position and demonstrates the highest degree of citation bursts.

The collisions of ChatGPT and medicine provide new insights. This study provides an overview of medical research articles pertaining to ChatGPT through bibliometric analysis, revealing research trends following the integration of ChatGPT with medicine. Notably, research topic hotspots closely tied to AI are continuously evolving, reflecting ChatGPT’s ongoing developmental potential. Concentrations of ChatGPT’s application are observed in areas such as digital health, clinical decision support, and large language models [9, 10]. However, it’s crucial to consider potential socioeconomic and cultural influences underlying these trends in future research. Furthermore, there is a need for deeper exploration into ChatGPT’s technical contributions to medicine. Additionally, the juxtaposition of ChatGPT with other AI tools like Gemini and Kimi. Exploring the synergies of combining various artificial intelligence tools such as DALL-E, DeepL, Typecast AI, and Resemble AI with ChatGPT allows for an investigation into their collective impact on medcine. This study enhances our understanding of ChatGPT’s research trends and topic hotspots in medicine, offering insights into current status and future directions.

## Electronic Supplementary Material

Below is the link to the electronic supplementary material.

Supplementary Material 1

## Data Availability

The original contributions presented in the study can be obtain from web of science or the corresponding author.
