# Supplementary material for: Publication Trends and Hot Spots of ChatGPT’s Application in the Medicine
Source: J Med Syst. 2024 May 18;48(1):52. doi: 10.1007/s10916-024-02074-y (PMC11102365; doi:10.1007/s10916-024-02074-y)
Supplement: Supplementary file 1 — Supplementary Material 1 [file 10916_2024_2074_MOESM1_ESM.docx]

# Publication Trends and hot spots of ChatGPT’s Application in the Medicine

eMethods 1. Definition of the metrics in Citespace Software

eMethods 2. Definition of the C-index and N-index

eTable 1.Top 5 countries/regions related to ChatGPT’s publications on medical

eTable 2.Top 5 institutions related to ChatGPT’s publications on medical

eTable 3.Top 5 author related to ChatGPT’s publications on medical

**eMethods 1**

**Definition of the index in Citespace Software**

CiteSpace is a cutting-edge bibliometric analysis software^1-5^, integral for delving into cooperative networks, co-occurrence matrices, and trends across a wide array of academic entities, including authors, institutions, and keywords. The essence of CiteSpace analysis is encapsulated in its scientific maps, characterized by nodes, links, and colors that vary in size and thickness, respectively. The larger the node, the higher the number of articles represented or the higher the frequency of co-occurrence. Distinctly colored annual rings around each node indicate the year of occurrence, aligning with a color legend for easy reference. A node with a purple outer ring signifies substantial centrality(node centrality>0.1), highlighting its critical role within the network. Links interconnecting nodes symbolize cooperative or co-occurrence relationships, with their thickness illustrating the strength of these connections. The color of links denotes the year they first appeared, with warmer colors representing more recent years. This visualization not only elucidates the structural dynamics of research networks but also accentuates the temporal evolution of research trends.

Co-occurrence network analysis visualizes the simultaneous appearance of different entities (such as countries, institutions, or authors) within the same or multiple documents to reveal relationships between them. These networks are depicted through nodes and links (co-occurrence relationships), with links weights typically based on the frequency of co-occurrences. Co-occurrence networks assist researchers in identifying major research trends, patterns, and potential interdisciplinary connections within a discipline. Furthermore, they facilitate the use of network analysis tools to explore the structure of academic fields, such as by calculating the centrality of nodes or identifying research clusters. Additionally, timeline visualizations organize clusters horizontally, mapping each from left to right according to publication dates, displayed at the visualization's bottom edge, the color-coded curves to denote co-citation connections formed within specific years, the large nodes or those with red halos—for their notable citation metrics or sudden increases in citations. Below these timelines, it showcases the year's top three cited works, positioning the highest-cited work at the bottom.

**eMethods 2**

**Definition of the C-index and N-index**

The C-index (Collaboration Index) is designed to evaluate the collaborative capability of researchers, departments, institutions, or any group with peer-level researchers across continents. It assesses collaboration in research publication across national and international boundaries. The key details about the calculation of the C-index without journal ranking were shown in previous research^6^. And N-index= highest h-index of the journals in the researcher’s field/ researcher’s h-index^7^.

**eTable 1.Top 5 countries/regions related to ChatGPT’s publications on medical**

| **Ranking** | Publications | Countries/regions | % of 574 | Centrality | C-index^a^ |
| --- | --- | --- | --- | --- | --- |
| **1** | 210 | USA | 36.585 | 0.16 | 179.9 |
| **2** | 59 | INDIA | 10.279 | 0.03 | 34.6 |
| **3** | 57 | CHINA | 9.930 | 0.00 | 32.1 |
| **4** | 55 | ENGLAND | 9.581 | 0.08 | 38.9 |
| **5** | 44 | GERMANY | 7.666 | 0.00 | 31.7 |

**eTable 2.Top 5 institutions related to ChatGPT’s publications on medical**

| Ranking | Publications | Institutions | % of 574 | Centrality | C-index |
| --- | --- | --- | --- | --- | --- |
| **1** | 23 | NATIONAL UNIVERSITY OF SINGAPORE | 4.007 | 0.03 | 12 |
| **2** | 21 | STANFORD UNIVERSITY | 3.659 | 0.07 | 10.4 |
| **3** | 19 | UNIVERSITY OF CALIFORNIA SYSTEM | 3.310 | 0.06 | 8.3 |
| **4** | 14 | HARVARD UNIVERSITY | 2.439 | 0.12 | 11.7 |
| **5** | 12 | TEL AVIV UNIVERSITY | 2.090 | 0.03 | 11.1 |

**eTable 3.Top 5 author related to ChatGPT’s publications on medical**

| Ranking | Author | Publications | % of 574 | Centrality | H-index | C-index | N-index |
| --- | --- | --- | --- | --- | --- | --- | --- |
| **1** | Wiwanitkit Viroj | 10 | 1.742 | 0.22 | 27 | 6.9 | 0.29 |
| **2** | Seth Ishith | 9 | 1.568 | 0.09 | 8 | 7.2 | 0.07 |
| **3** | Klang Eyal | 7 | 1.220 | 0.07 | 30 | 7 | 0.10 |
| **4** | Kleebayoon Amnuay | 7 | 1.220 | 0.07 | 4 | 6.4 | 0.05 |
| **5** | Rozen WM | 6 | 1.045 | 0.05 | 45 | 4.4 | 0.28 |

**Reference**

1.Ninkov A, Frank J R, Maggio L A. Bibliometrics: Methods for studying academic publishing. Perspectives on Medical Education, 2021, 11(3): 173–17

2.Ye-na Gan, Duo-duo Li, Nicola Robinson et al. Practical guidance on bibliometric analysis and mapping knowledge domains methodology-A summary, European Journal of Integrative Medicine.Volume 56, 2022,102203.https://doi.org/10.1016/j.eujim.2022.102203.

3.D. University, U.C. Yue Citespace Ⅱ: detecting and visualizing emerging trends and transient patterns in scientific literature. J Assoc Inf Sci Technol, 57 (3) (2014), pp. 359-377

4.Synnestvedt MB, Chen C, Holmes JH. CiteSpace II: visualization and knowledge discovery in bibliographic databases. AMIA Annu Symp Proc. 2005;2005:724-8.

5.Fonseca, B.d., Sampaio, R.B., Fonseca, M.V.d. et al. Co-authorship network analysis in health research: method and potential use. Health Res Policy Sys 14, 34 (2016). https://doi.org/10.1186/s12961-016-0104-5

6. Chattopadhyaya, Somnath, Alam, Firoz, Chowdhury. Harun. “A novel C-index for evaluation of research collaboration.”AIP Conference Proceedings vol. 2681, 1(2022): 020096. doi:10.1063/5.0117099.

7. Namazi MR, Fallahzadeh MK. n-index: a novel and easily-calculable parameter for comparison of researchers working in different scientific fields. Indian J Dermatol Venereol Leprol. 2010 May-Jun;76(3):229-30. doi: 10.4103/0378-6323.62960.
